# Molecular Structure of Cefuroxime Axetil Complexes with α-, β-, γ-, and 2-Hydroxypropyl-β-Cyclodextrins: Molecular Simulations and Raman Spectroscopic and Imaging Studies

**DOI:** 10.3390/ijms22105238

**Published:** 2021-05-15

**Authors:** Barbara Gieroba, Grzegorz Kalisz, Anna Sroka-Bartnicka, Anita Płazińska, Wojciech Płaziński, Małgorzata Starek, Monika Dąbrowska

**Affiliations:** 1 Department of Biopharmacy, Medical University of Lublin, ul. Chodzki 4a, 20-093 Lublin, Poland; grkalisz@gmail.com (G.K.); anna.sroka@umlub.pl (A.S.-B.); anita.plazinska@umlub.pl (A.P.); 2 Department of Genetics and Microbiology, Institute of Microbiology and Biotechnology, Maria Curie-Skłodowska University, ul. Akademicka 19, 20-033 Lublin, Poland; 3 Jerzy Haber Institute of Catalysis and Surface Chemistry, ul. Niezapominajek 8, 30-239 Krakow, Poland; 4 Department of Inorganic and Analytical Chemistry, Faculty of Pharmacy, Jagiellonian University Medical College, ul. Medyczna 9, 30-688 Kraków, Poland; m.starek@uj.edu.pl (M.S.); monika.1.dabrowska@uj.edu.pl (M.D.)

**Keywords:** cefuroxime axetil, cyclodextrins, drug delivery, inclusion complexes, Raman spectroscopy, molecular dynamics

## Abstract

The formation of cefuroxime axetil+cyclodextrin (CA+CD) complexes increases the aqueous solubility of CA, improves its physico-chemical properties, and facilitates a biomembrane-mediated drug delivery process. In CD-based tablet formulations, it is crucial to investigate the molecular details of complexes in final pharmaceutical preparation. In this study, Raman spectroscopy and mapping were applied for the detection and identification of chemical groups involved in α-, β-, γ-, and 2-hydroxypropyl-β-CD (2-HP- β-CD)+CA complexation process. The experimental studies have been complemented by molecular dynamics-based investigations, providing additional molecular details of CA+CD interactions. It has been demonstrated that CA forms the guest–host type inclusion complexes with all studied CDs; however, the nature of the interactions is slightly different. It seems that both α- and β-CD interact with furanyl and methoxy moieties of CA, γ-CD forms a more diverse pattern of interactions with CA, which are not observed in other CDs, whereas 2HP-β-CD binds CA with the contribution of hydrogen bonding. Apart from supporting this interpretation of the experimental data, molecular dynamics simulations allowed for ordering the CA+CD binding affinities. The obtained results proved that the molecular details of the host–guest complexation can be successfully predicted from the combination of Raman spectroscopy and molecular modeling.

## 1. Introduction

**Figure 1:**
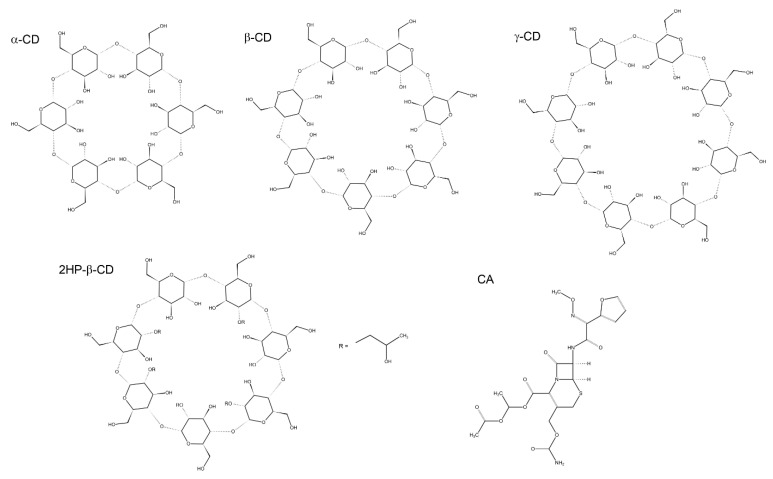
Chemical structures of cefuroxime axetil (CA) and the most common natural cyclodextrins: α-, β-, and γ-CDs as well as 2-hydroxypropyl-β-cyclodextrin (2HP-β-CD). The functionalization pattern of 2HP-β-CD was accepted according to the compound used in the present study.

Cyclodextrins (CDs), discovered by Antoine Villiers in 1891 [1], are oligosaccharides that consist of D-glucopyranose units linked via α(1→4)-bonds and forming a cyclic structure. The restricted rotation around glycosidic linkage and a cone-like construction with a lipophilic central interior and hydrophilic external surface make them less prone to degradation by enzymes in comparison to dextrins with linear arrangement [2]. Among commonly appearing natural CDs, α-CD, β-CD, and γ-CD can be distinguished on the basis of the number of glucose subunits (six, seven and eight subunits, respectively) [3]. They possess numerous primary hydroxyl and hydroxymethyl groups on the edges of the entries to the inner channel, serving as active sites to create a vast range of derivatives and linkages (Figure 1).

CDs show a great potential to form inclusion complexes by non-covalent, attractive intermolecular interactions, such as hydrogen bonds and van der Waals’ forces, with numerous molecules by incorporating them in their inner hydrophobic cavity and without affecting their biological, chemical and physical characteristics [4,5]. This remarkable encapsulation has the nature of a “host-guest” type relationship, which, from the point of view of the complexed guest, increases its solubility, enhances bioavailability and stability, decreases unwanted adverse effects, prevents drug–drug and drug–excipient interactions (these properties are based on reduction of the free drug, which also diminishes gastrointestinal, ocular and rhinal irritations [6]), improves absorption across biomembranes, and extends the release of the encapsulated molecule, expanding the spectrum of application. Additionally, in anticancer treatment, the rate-controlled release of 5-fluorouracil from its complex with β-CD decreases systemic side-effects and provides an effective therapy with reduced dose and shortened time [7]. These features make CDs useful in drug delivery [8]. α-, β-, and γ-CD are all generally recognized as safe, having the “GRAS status” attributed by the U.S. Food and Drug Administration (FDA) [9]. The multifunctional properties of CDs have empowered them to be applied in nearly all drug delivery systems, such as oral, sublingual, rectal, nasal, pulmonary, ophthalmic, dermal and transdermal [10,11]. Moreover, studies with CD derivatives, mainly HP-β-CD, suggest that they have no adverse effects on the kidneys after both oral and intravenous administration [12]. It is also worth mentioning that CDs may act as an artificial circulating carrier for the lipophiles to redistribute them in the extracellular space, reducing unwanted effects. Some natural and exogenous lipophiles are becoming toxic for a prolonged time when the organism is not able to transport and redistribute them properly. This results in their accumulation in fat tissues, where they are not metabolized effectively. This is prevented by the formation of lipophilic drug–CD complexes [13]. They also are implemented in the design of some new delivery systems, such as liposomes, microspheres, microcapsules, nanoparticles, hydrogels, nanosponges and nanogels, beads, and finally CD-containing polymers [11,14]. Besides the pharmaceutical application, they are used in food processing, agriculture, and environmental and chemical engineering (especially in the field of chromatography, catalysis, biotechnological processes, and enzyme technology). Moreover, they are utilized in other branches of industry, such as cosmetics, aromas, hygiene, medicine, and textiles [1]. Selected properties of cyclodextrins are summarized in Table 1.

The hydroxyalkyl derivative of β-CD, 2-hydroxypropyl-β-cyclodextrin (2HP-β-CD), is an alternative with respect to α-, β- and γ-CDs, with improved water solubility properties and greater stability [20]; therefore, it finds application as the most versatile excipient formulation vehicle among the cyclic oligosaccharides used for the prosperous delivery of medical agents to biological systems [21,22]. Furthermore, numerous studies showed that 2HP-β-CD is toxicologically safe and well tolerated in human organisms [23]. Recently, growing interest is directed towards pharmacologically active, chemically modified CDs, in particular hydroxypropylated (which has been examined in this study), methylated, acetylated, carboxymethylated, sulfobutyl-ether and branched, such as glycosylated, glucoronylglycosyled or maltosylated cyclodextrin derivatives. Thus, the breadth of CD applications will probably increase quickly in the near future [10].

In the pharmaceutical formulation studies, CDs have mostly been applied as complexing compounds to increase the water solubility and to improve the bioavailability and stability of poorly soluble drugs, for example, cephalosporins [24], including cefuroxime. Cefuroxime axetil (CA), an 1-acetoxyethyl ester of cefuroxime (prodrug), is a broad spectrum second-generation semisynthetic cephalosporin comprising a β-lactam ring [25], being effectively absorbed from the gastrointestinal tract due to its lipophilicity and subsequently de-esterified to cefuroxime [26]. The antibacterial activity of cefuroxime in vivo is the result of its capacity to bind target proteins located in the bacterial cell wall, which leads to the inhibition of cell wall synthesis, hence the bacteria lose their ability to divide and mature. CA is a selective inhibitor of peptidoglycan synthesis characterized by good stability against bacterial β-lactamases [27,28]. Apart from treating lower and upper respiratory tract infections, CA may also be used in the therapy of skin and soft tissue infections, genitourinary tract bacterial infections and prophylactically in coronary artery bypass grafting surgery or cholecystectomy [29]. The wide range of cefuroxime activity includes Gram-positive bacteria: *Streptococcus pneumoniae* strains (with reduced penicillin susceptibility), *Streptococcus pyogenes* and *Staphyloccocus* strains (methicillin-sensitive strains), as well as Gram-negative strains of *Haemophilus influenzae*, *Moraxella catarrhalis*, *Klebsiella pneumoniae*, *Proteus mirabilis*, *Escherichia coli* and *Neisseria meningitis*. The presence of an iminomethoxy group in the acyl side chain significantly increases the stability of cefuroxime against β-lactamases found by *H. influenzae*, *Neisseria gonorrhoeae* and some *Enterobacteriaceae* [30]. In the Biopharmaceutics Classification System (BCS), CA is a class II drug (poorly soluble/highly permeable) and possesses only 55% absolute oral bioavailability [31], which justifies its coupling with CDs, improving water solubility and cellular internalization [32,33].

Raman spectroscopy is one of the most commonly and widely used analytical methods, applied mostly in inorganic, organic, and physical chemistry as well as in biology, pharmacy, and biomedical sciences due to its convenient appliance in identifying and determining biochemical structures of both single compounds and their mixtures [34]. This vibrational technique enables specific, cost-effective and non-destructive real-time measurements with high sensitivity, low limit of detection and no need for sample staining and special preparation. For this reason, it is often referred to as a “green analytical method”. Furthermore, it requires a small amount of studied material with high reproducibility of fast collected data and, what is equally important, the spectrometers are facile to operate and maintain [35]. The combination of Raman spectroscopy with microscopy can provide advantageous information on spatial distribution and homogeneity both within and between samples containing individual substances and their complexes and/or conjugates (pharmaceutical systems) at a microscopic level with high resolution [36]. To confirm the Raman spectroscopic data, additional methods providing information on the molecular structure of the guest–host inclusion CD+CA complexes, such as fluorescence and spectrophotometric techniques, Fourier Transform Infrared (FT-IR) and Nuclear Magnetic Resonance (NMR) spectroscopy, Scanning Electron Microscopy (SEM), Differential Scanning Calorimetry (DSC), and X-ray diffractometry, would be very helpful [37].

There exist several examples of studies describing various types of CD+CA inclusion complexes. Mizera et al. studied the solubility and antimicrobial activity of CA in systems, prepared with a co-precipitation method in 1:1 (CA:CD) molar ratio, designed according to the guidance of a validated in silico model [38]. Other studies were focused on the investigation of the physicochemical properties of the binary and ternary inclusion complexes of CA with βCD in the presence of L-arginine, prepared by the spray drying method [39]. The physicochemical properties of spray dried microcomplexes of CA with HP-β-CD in the presence of various components (polyvinylpyrrolidone K30, hydroxypropylmethylcellulose, poloxamer 188, polyethylene glycol 4000, Aerosil 200) were investigated [40]. The available manuscripts relate essentially to the study of drug behavior in complexes with CD/CDs (also in the presence of co-existing substances, e.g., arginine) formed by co-dissolution. Our research includes a comprehensive analysis of CA behavior in complexes with four CDs, formed in the process of mechanical complexing by tableting. We will check how this method affects the quality of the created CD+CA connections, by identifying individual groups responsible for the intermolecular interactions. In our opinion, this study is an important step forward in the understanding of the exact molecular mechanism which is involved in the formation of an inclusion complex between CA and selected CDs. Our approach is more structural (using experimental, computational and simulation methods) and less biological, which has been the main focus of researchers so far.

The purpose of the present research was the qualitative and semi-quantitative detection of functional groups and determination of their vibrational modes in cefuroxime axetil–cyclodextrin (α-, β-, γ-CD and 2HP-β-CD) complexes, which reflect the molecular arrangement, chemical moieties participating in the complexation together with their share, and type of interactions involved in the complex formation. In order to obtain these data, we have applied vibrational Raman spectroscopy combined with molecular dynamics simulations at atomic resolution. The Raman spectroscopy allowed for the detailed physico-chemical analysis of the CD+CA conjugates with a special focus on the surface property characterization. Additionally, to examine the spatial distribution on the surface of the tablet of particular chemical groups, Raman mapping was performed. The research enabled the examination of the differences in the molecular conformation between the studied CD+CA complexes, which may be relevant for their pharmaceutical application, i.e., for the controlled drug delivery and entrapment ensuring sustained, prolonged or rapid release of the drug depending on their characteristics. Additionally, the molecular dynamics simulations were performed to study the molecular details of interactions between CA and CD in the corresponding conjugates as well as to estimate the strength of the CD+CA binding, expressed as the free energies of binding.

The results allowed us to elucidate the molecular details of the binding of CA to various CDs, including the identification of the most crucial intermolecular interactions. In particular, we have found that 2HP-β-CD is the most suitable compound to be considered for CA encapsulation. Finally, the potential and synergic effect of combined molecular dynamics simulations with Raman spectroscopic studies in the case of investigating the CD–guest systems is demonstrated.

## 2. Results

### 2.1. Raman Spectroscopy

**Figure 2:**
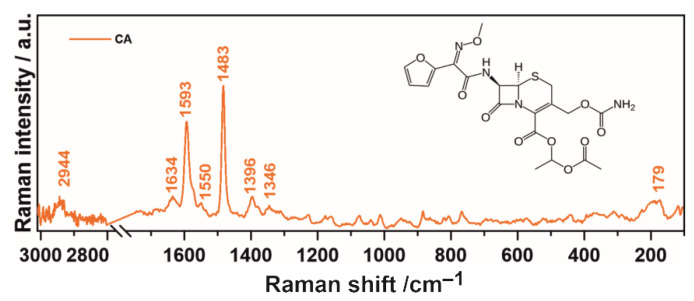
The Raman spectrum and the chemical structure of cefuroxime axetil.

The analysis of the Raman spectrum of pure CA in the tablet form is presented in Figure 2. It confirms its identification and is compatible with literature reports [41,42]. Characteristic bands detected at 1593 cm^−1^ can be attributed to C=C stretching in the furanyl ring and C=N stretching vibrations in the (methoxyimino) acetyl group specific to the amorphous form of CA, bands at 1483 cm^−1^ are assigned to C=C stretching in the furanyl ring and C–C stretching, N–H, and CH_2_ scissoring modes (in CH_3_ group) between the furanyl ring and NOCH_3_ group in the (methoxyimino)acetyl group occurring both in the amorphous and crystalline form of CA, bands at 1396 cm^−1^ are connected with CH_3_ scissoring in the (acetyloxy)ethyl group and bands at 1346 cm^−1^ are ascribed to C–O, C–N, and CH_2_ stretching modes in the furanyl(methoxyimino)acetyl group. Other recorded bands at 2944, 1634, 1550 and 179 cm^−1^ are assigned to CH_3_ antisymmetric stretching, C=C stretching, C–C stretching vibrations and skeletal deformations, respectively [41,42].

**Figure 3:**
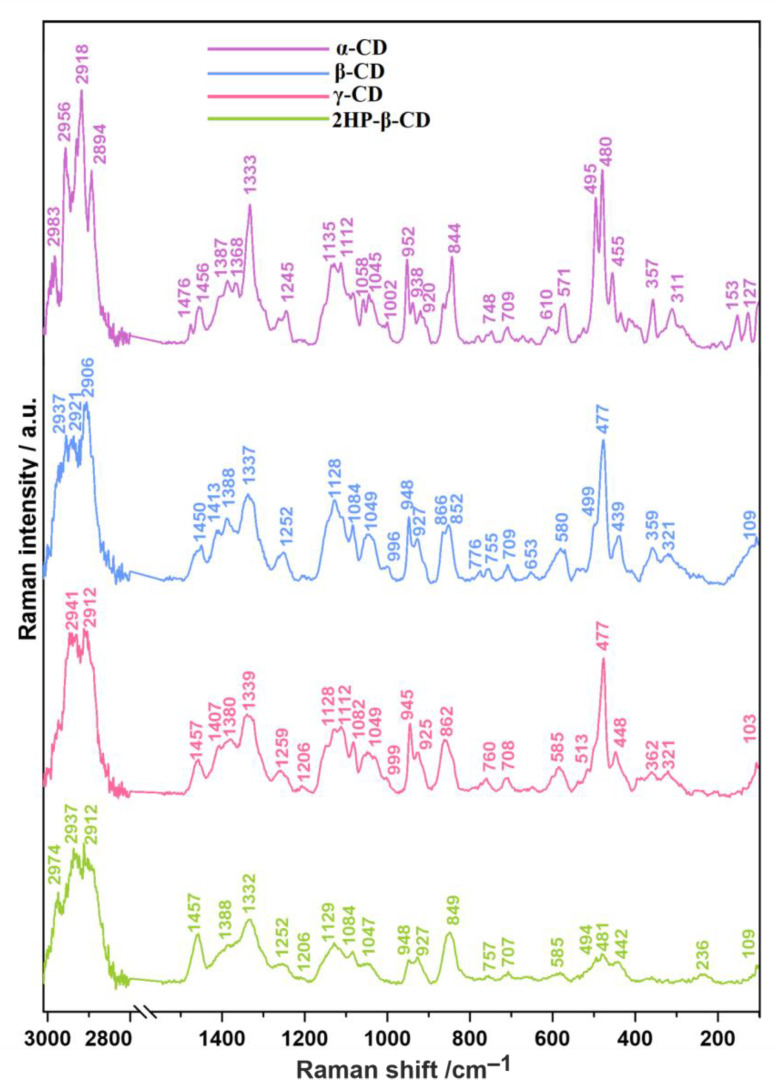
The Raman spectra of pure α-, β-, γ- and 2HP-β-CDs.

The spectra of α-, β-, γ- and 2HP-β-CDs (Figure 3) are also consistent with the literature data and allow for the undisputed identification of these compounds [43]. Briefly, the most important bands are those extending from 3000 to 2700 cm^−1^, describing the C–H stretching vibrational mode, bands ranging from 1500 to 1000 cm^−1^ related mainly to the C–C and C–O stretching vibrations, bands in the 900−600 cm^−1^ region, connected with bending modes of C–H bonds, and finally the bands in the 600–400 cm^−1^ spectral range assigned to macrocyclic ring deformational modes [43].

**Figure 4:**
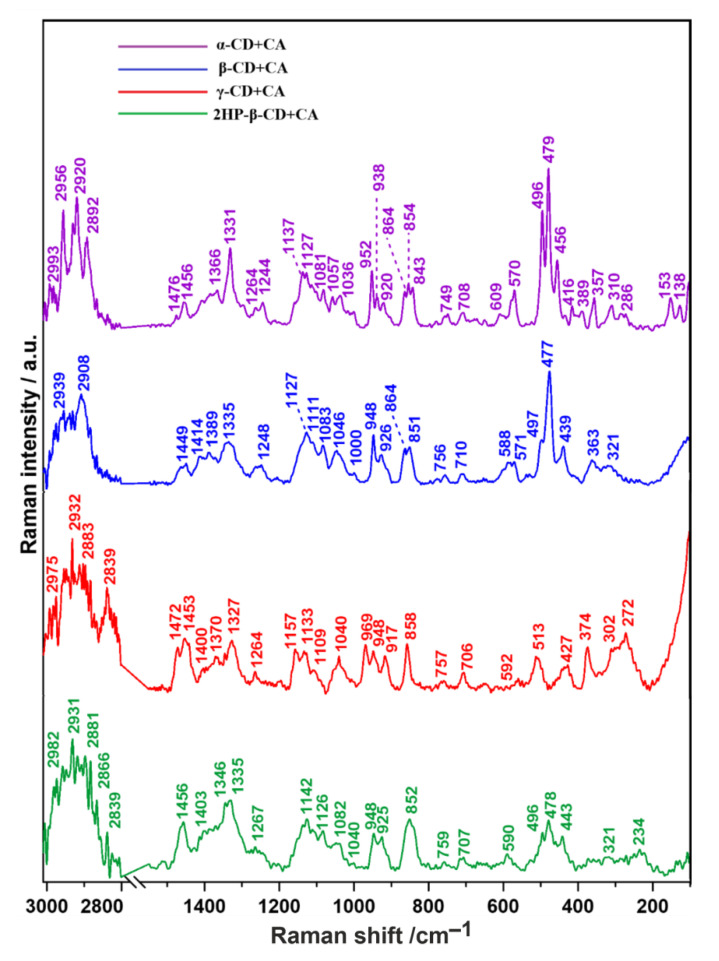
Raman spectra of inclusion complexes of cefuroxime axetil with α-, β-, γ-CDs and 2HP-β-CD along with band assignments.

Raman spectra of tablets containing complexes of all studied CDs with cefuroxime axetil are depicted in Figure 4. The predominant bands obtained in Raman spectra of examined samples and their attribution to specific chemical groups are summarized in Table 2.

The significant bands assigned to stretching vibrations of CH_3_, C–C and C–H recorded in the 1600–1500 cm^−1^ range of the CA Raman spectrum disappeared after association with all types of CDs. The above-mentioned observation suggests the involvement of the corresponding moieties in interactions between CA and CDs. The different intensities and values of Raman shifts of carbonyl (C=O), C–O bonds and CH_3_ groups may mean the formation of hydrogen bonds. Together with the appearance of the imino C–N and CNC bands and C–H substituents in the β-lactam ring, these changes may indicate the formation of CA–CD host–guest inclusion complexes [48]. The spectra of pure CDs and CA–CD complexes are very similar; they differ only in minor frequency shifts and variations in relative intensities. These observations can also confirm the non-covalent inclusion of the CA molecules inside the cyclodextrin cavity. However, the non-uniform character of spectral alterations suggests that the modes of interaction between the particular CDs and CA are slightly different. The most crucial differences between studied systems are marked in Table 2 with an asterisk (*).

The band located at ~477–480 cm^−1^ is ascribed to the skeletal mode of the pyranose ring [49]. In fact, in the spectrum of pure 2HP-β-CD it is much less intense, broadened and shifted to a higher frequency (481 cm^−1^), but is still present. Fechner, Siegfried, Kleinebudde, and Reinhard (2005) proved that the position of this band changes in intensity as a function of the amount of amylose present in the sample [50]. Here, it may result from the masking of pyranose units by externally arranged hydroxypropyl moieties. However, it completely disappears in the γ-CD+CA complex, but in this case the band at 917 cm^−1^ attributed to glucopyranose (C–O–C) skeletal mode of α-anomers is detected. This fact may probably be due to the superimposition of the pyranose ring vibration and the asymmetric stretching vibration of the glycosidic C–O–C bonds [51].

In the case of the α-CD+CA complex, additional bands related to CH and CH_3_ stretching vibrations appear. Additionally, bands associated with the breathing mode of oxygen vibration in the macrocyclic ring and hydrogen bonds emerge. It is worth noting that, additionally, there appear bands resulting from the CA C–C–C ring in-plane bending, indicating the substantial participation of the furanyl ring of CA in the formation of complexes.

Analyzing the spectrum of β-CD+CA, the appearance of bands associated with external C–OH out-of-plane bending of glucopyranose units, C=C–H stretching vibrations, the “breathing mode” of the aromatic carbon ring, and different patterns of C–O–C stretching bands suggests the significant involvement of the inner surface of the macrocyclic ring of β-CD.

Considering the spectrum of γ-CD+CA, there are some similarities to the α-CD+CA complex, such as the presence of bands assigned to OH, C–C, and C–O stretching. In addition, the bands attributed to ring deformation and –C=C–H antisymmetric angular deformation in plane arise, which may imply both the participation of the furanyl ring of CA and the close contact of the inner surface of the macrocyclic ring of γ-CD with the condensed β-lactam rings of CA. This strict interaction is also confirmed by the loss of the band at 477 cm^−1^ present in the spectrum of pure γ-CD.

In the 2HP-β-CD+CA spectrum, new bands related to deformations of the CH_2_OH group, and C–O, C–N stretching modes reveal, pointing to the special commitment of the furan, hydroxymethyl and imine moieties in this type of complex.

**Figure 5:**
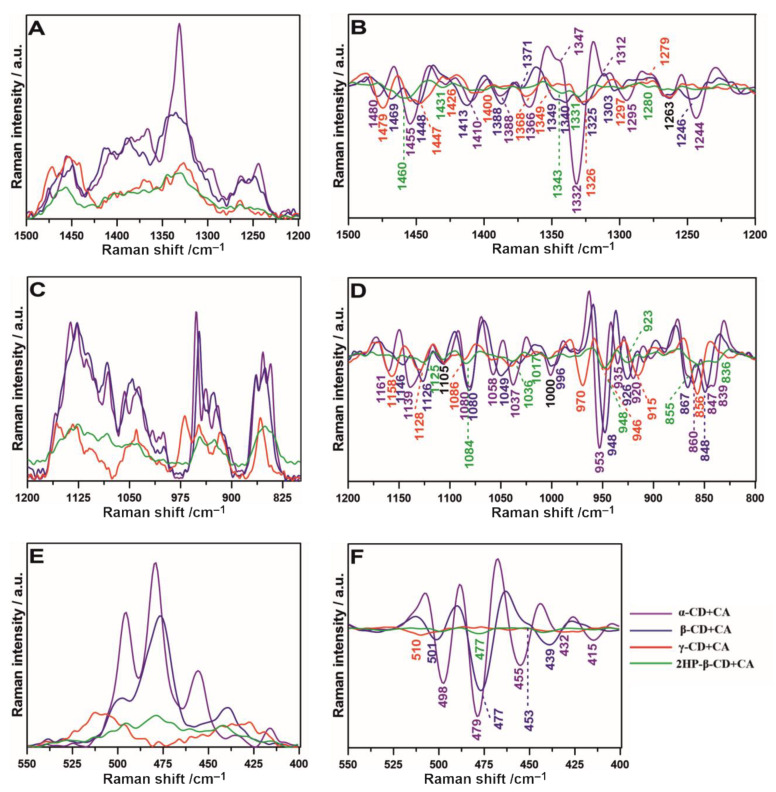
The relative intensities of Raman spectra and their second derivatives in the selected ranges: 1500–1200 cm^−1^ ((**A**,**B**), respectively), 1200–800 cm^−1^ ((**C**,**D**), respectively), and 550–400 cm^−1^ ((**E**,**F**), respectively). Numbers in black indicate the same Raman shift values for all samples.

As measured Raman spectra showed that each of the considered CDs complexes with CA in a diverse manner, we have determined the second derivative of the spectra (Figure 5) in the three ranges: 1500–1200 cm^−1^, helpful for the detailed assessment of CH_2_OH, CH, CH_2_ and CH_3_ stretching and deformational modes; 1200–800 cm^−1^, corresponding to the spectral region of polysaccharides (principally stretching vibrations of C–O/C–C groups) [52]; and 550–400 cm^−1^, connected with skeletal and ring deformation, C–C–C bending, and OH and C–C stretching [47].

The relative intensity spectra recorded for α-CD+CA and β-CD+CA are characterized by increased signal compared to those obtained for γ-CD+CA and 2HP-β-CD+CA (Figure 5A,C,E). Observing the course of the second derivative, the number of similarities between α-CD+CA and β-CD+CA in all studied ranges can be found; the results differ mainly in small shifts (Figure 5B,D,F). In the 1500–1200 cm^−1^ range (Figure 5B) for α-CD+CA, the appearance of second derivative minima at 1455, 1322, and 1244 cm^−1^ is clearly visible and can be assigned to both CH and CH2 bending, wagging/twisting, and stretching modes [53], respectively. In all spectra, the band at 1263 cm^−1^ attributed to C–O scissoring [54] is present at the same Raman shift. In the 1200–800 cm^−1^ range (Figure 5D), the appearance of the band at 970 cm^−1^ arising from C–H deformational mode in the γ-CD+CA spectrum is noteworthy; also, the bands at 1058 and 1037 cm^−1^ in β-CD+CA and 1049 cm^−1^ in α-CD+CA connected with C–O stretching [55] testify to the differences in the structural organization in this type of association. Considering the 550–400 cm^−1^ region (Figure 5F), the band at 510 cm^−1^ appears only for γ-CD+CA, which is related to C–C–O bending and C–C and C–O stretching vibrations [56], while the band at ~477 cm^−1^ assigned to bending C–C–C and twisting C–O vibrations, characteristic for polysaccharides, especially amylose [57], is barely pronounced. At the same time, it is much more explicit in the α-CD, β-CD, and 2HP-β-CD+CA samples. It may mean that γ-CD interacting with CA forms a variety of distinct interactions that are not detected in the case of other studied CDs. This is probably the result of the larger size of the inner cavity of γ-CD and deeper insertion of CA inside this cavity. In turn, the detection of various vibrational modes of the CH group in α-CD+CA indicates the involvement of furan groups because furan contains the five-membered aromatic ring, consisting of four CH groups accompanied by one oxygen atom [58].

**Figure 6:**
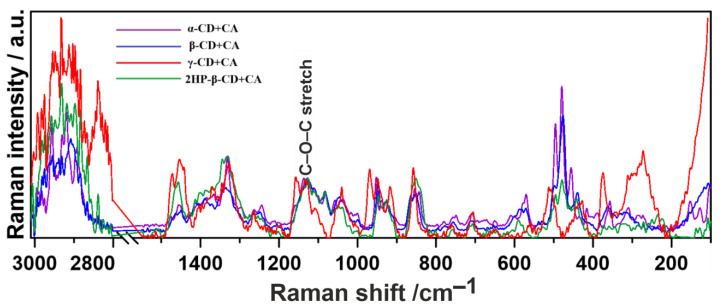
Raman spectra normalized to 1127 cm^−1^ (α-CD+CA and β-CD+CA), 1133 cm^−1^ (γ-CD+CA) and 1126 cm^−1^ (2HP-β-CD+CA) bands.

Based on the Raman spectra, the molecular arrangement and the manner of interactions in chemical complexes can also be assessed semi-quantitatively by calculations of the Raman intensity ratio parameters for the most important chemical groups involved in the CD inclusion and complexation processes, selected on the basis of the reported data [59,60]. The compilation of obtained results is presented in Table 3. Before the analysis, the spectra were normalized to the C-O-C stretching vibration bands, i.e., those appearing at 1127 cm^−1^ (α-CD+CA and β-CD+CA), 1133 cm^−1^ (γ-CD+CA) and 1126 cm^−1^ (2HP-β-CD+CA) (Figure 6).

The Raman intensity ratio enables us to carry out a quantitative analysis of molecular mechanisms of interactions. Evidently lower I_OH/CH2_ and higher I_C=O/OH_ coefficient values in the 2HP-β-CD+CA complex indicate that the OH groups are located rather in the external cavity area and do not participate in the inclusion process. The I_C=O/CH2_ ratio is similar in α-CD+CA and 2HP-β-CD+CA and also in β-CD+CA and γ-CD+CA samples. The I_OH/CH2_ factor is comparable in all specimens except 2HP-β-CD+CA. The quite high I_C=O/OH_ ratio in the γ-CD+CA sample is also noteworthy. Considering these data, we can state that for both α- and β-cyclodextrins, the formation of CA complexes occurs according to very comparable mechanisms and γ-CD+CA complexes have a slightly different character, indicating greater involvement of carbonyl rather than hydroxyl groups in complex formation, while the greatest differences are characteristic of 2HP-β-CD+CA mainly due to dissimilar distribution of OH moieties.

**Figure 7:**
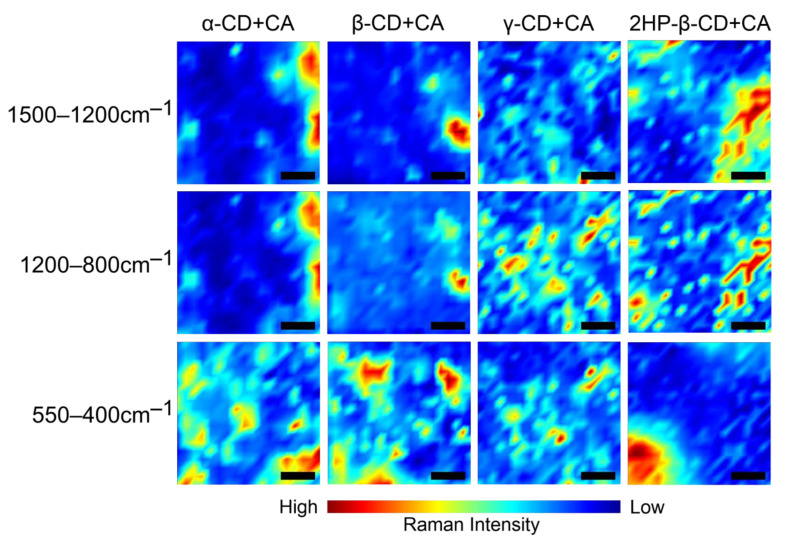
Raman chemical maps of structural moiety distribution in CA–CD complexes. Black scale bar at the bottom of the images corresponds to 20 µm.

In the next stage, we performed Raman spectroscopic imaging of the surface distribution of the most crucial chemical groups within previously studied ranges: 1500–1200 cm^−1^, 1200–800 cm^−1^ and 550–400 cm^−1^ of CD+CA complexes (Figure 7). The assignments are explained in the description of Figure 4.

Raman spectroscopy and microscopy have been used to investigate the distribution of different chemical components at the surface of studied CD+CA tablets. The visualization (Figure 7) depicts the spatial distribution and Raman intensity of the signal of the most important chemical compounds in the xy-plane of the sample images. Differences in the composition of studied complexes indicate a distinct manner of creating molecular connections. γ-CD+CA and 2HP-β-CD+CA show a higher intensity in 1500–1200 cm^−1^ and 1200–800 cm^−1^ ranges with greater homogeneity of distribution and a lower intensity in the 550–400 cm^−1^ range with less homogeneity than α-CD+CA and β-CD+CA samples. This may be due to the fact that the larger inner dimensions of the cavity of γ-CD favor the deeper incorporation of the CA into the macrocyclic ring structure, forming multiple interactions that are not observed in α- and β-CDs with smaller inner cavity dimensions. In the case of 2HP-β-CD+CA, the similar result may be due to the presence of additional CH_2_–CH(OH)–CH_3_ groups, which may participate in creating different patterns of the hydrogen bond network. Imaging revealed that in both α-CD+CA and β-CD+CA complexes, the nature of interactions is quite similar.

**Figure 8:**
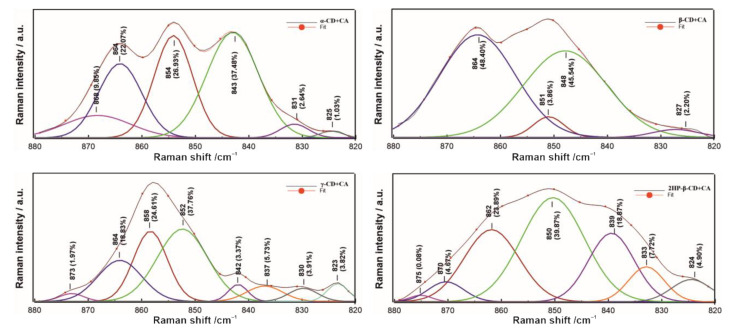
Deconvolution of 880–820 cm^−1^ band into particular sub-bands using mixed Lorentzian/Gaussian curve fitting. Each sub-band has marked a maximum value and a percentage share of its entire band surface area.

To further understand the nature of the interactions between CA and individual CDs, we performed a band deconvolution in the range of 880–820 cm^−1^ (Figure 8), attributed to C–C skeletal, C–O–C and CNC stretching vibrations [47,61].

At first glance, the band in the 880–820 cm^−1^ spectral region has a different course for all CD–CA systems (Figure 8); in the case of α-CD+CA, this band has three characteristic peaks, whereas it is quite narrow in the case of γ-CD+CA, and broadened both for β-CD+CA and 2HP-β-CD+CA. The smallest number of components is included in the band for β-CD+CA (four), and the larger in that for γ-CD+CA (eight). The 875–873 cm^−1^ sub-bands detected only for γ-CD+CA and 2HP-β-CD+CA may indicate additional interactions with water molecules and the external placement of hydroxyl groups [62], respectively. In the case of β-CD+CA, no bands in the 880–870 cm^−1^ and one band with a small contribution in 840–820 cm^−1^ regions were detected; the latter range is ascribed to the symmetric stretching mode of the CNC groups [63], where this share is much greater in comparison to other studied systems. The 843 cm^−1^ extensive sub-band in α-CD+CA and 842 cm^−1^ small sub-band in the γ-CD+CA spectrum are correlated with C-C and C-O stretching modes [52]. The 854–850 cm^−1^ bands are characteristic for the C-O-C skeletal mode of α-glycosidic bonds [64]; their smallest share was found for β-CD+CA.

### 2.2. Molecular Modeling

**Figure 9:**
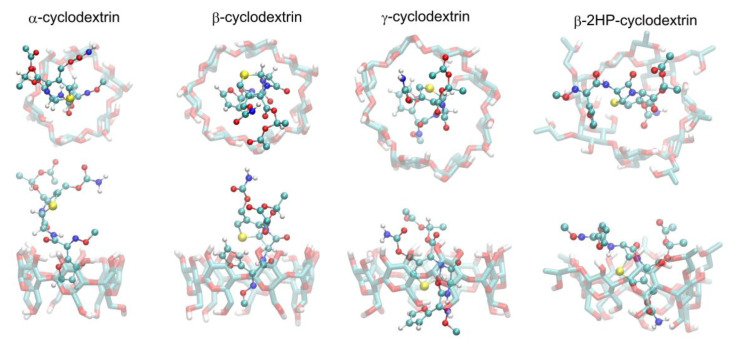
The MD simulation snapshots, illustrating the patterns of interactions typical for the studied complexes. Guest (CA) molecule is shown by an opaque ball-and-stick representation, while host (CD) molecule is represented by a transparent stick representation. The two views of each complex along perpendicular axes are shown.

**Figure 10:**
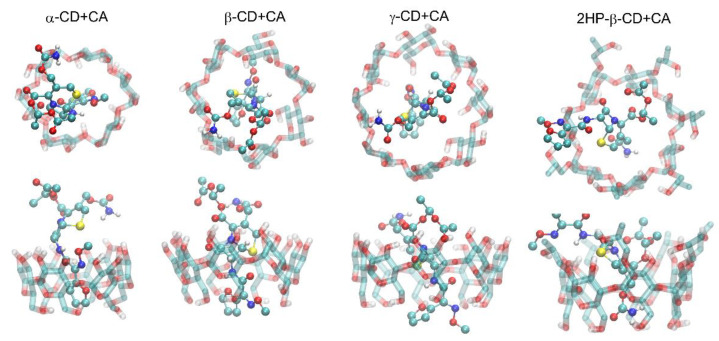
The most favorable structures of the CA–CD complexes identified in the docking simulations. The rest of details as in Figure 9.

Both types of initial arrangements of each of the cyclodextrin-containing systems (i.e., either disconnected CA and CD molecules or the complexes prepared on the basis of information from ref. [39]) lead to the same type of dynamic complex, which is strong evidence that the final structures really represent the thermodynamic equilibrium and can be used in the subsequent free energy calculations. Moreover, these equilibrium structures are compatible with the results of the docking simulations, which is another piece of evidence for the correctness of the obtained structures of the CA–CD complexes. The graphical illustration of the obtained complexes is given in Figure 9 and Figure 10. In all cases, the binding process is driven by the interactions of the guest (cefuroxime axetil) molecule with the inner surface of the macrocyclic ring of the cyclodextrin (host) molecule. The results of docking have only a character of reference for MD simulations, thus, only the latter approach will be discussed in detail. It is also worth noting that the most significant difference between MD- and docking-derived binding modes (observed in the case of β-CD) disappears during the course of the MD simulation when initiating it from the docking-predicted pose.

For both α- and β-CDs, the binding of CA has a very similar character. The moiety involved in the guest/host contact in the largest extent when considering binding to α-CD is the furan ring. This type of contact is limited to the inner surface of the macrocyclic ring of cyclodextrin. Additionally, the groups in the vicinity of the furanyl ring exhibit contact with the hydroxyl groups at the edge of the CD cavity. Due to the limited size of this ring, the remaining parts of the guest molecule do not show any intensive interactions with the host molecule but maintain interactions with surrounding water molecules.

In the case of β-CD, the additional *N*-methoxy and carbonyl moieties are involved in the interactions with the inner pocket of CD, which reflects the increased size of the cyclodextrin cavity. Interestingly, the furan–cyclodextrin interaction pattern is notably modified in comparison to that observed for α-CD; the cyclodextrin binding site is not large enough to simultaneously and fully accommodate both these moieties, thus, the furan ring is reoriented toward the main part of the ligand molecule, exhibiting relatively large distance from the methoxy group. Finally, an additional carbonyl group displays contact with hydroxyl groups at the edge of the CD cavity.

In comparison to α- and β-CDs, binding to γ-CD has a more intimate character. The larger dimensions of the binding cavity facilitate the deeper immersion of the CA molecule into the macrocyclic ring and the creation of a series of interactions that are not present in the case of smaller-size α- and β-CDs. More precisely, the inner surface of the macrocyclic ring of γ-CD maintains close contact with the condensed rings of CA, including the β-lactam moiety. At the same time, the furan and methoxy moieties, identified as essential in binding to α- and β-CD, do not exhibit intensive contact with the inner channel part of the γ-cyclodextrin molecule; instead, they freely interact with water molecules and hydroxymethyl moieties at the second edge of the CD inner channel. The latter can also be stated with regard to the amide moiety of CA and the second edge of the CD cavity.

Finally, binding to 2HP-β-CD results in the formation of a complex of different molecular topology, in comparison to un-functionalized cyclodextrins. Namely, the guest molecule is reoriented in such a way that the condensed ring cluster interacts with the hydrophobic patches at the inner cyclodextrin channel, whereas the amide group is immersed deeply into the channel which enables its interactions with hydroxymethyl groups of the host molecule. The remaining groups of CA are involved in the contact with 2-hydroxypropyl groups, attached at the edge to the cyclodextrin channel. These interactions have a character of both hydrogen bonding and non-polar contacts.

In the case of all considered complexes, their large conformational flexibility is associated with frequent reorientations of the guest molecule. This results in a highly dynamic structure and variable interaction patterns. Thus, the above description has qualitative rather than quantitative character and is focused on the most representative patterns of interactions.

**Figure 11:**
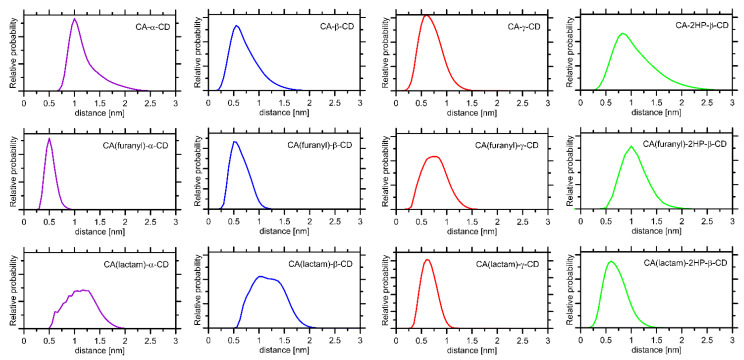
The radial distribution functions (RDFs) calculated from the MD simulations for selected parts of the system. They included pyranose rings of CDs (center-of-mass) and either the whole CA molecule (upper panels) or some of the functional groups present in the CA molecules and identified as significant in CA–CD binding.

The above-discussed qualitative results can be expressed in a more quantitative manner by considering the radial distribution functions (RDFs) calculated from the unbiased MD trajectories for each of the considered complexes (Figure 11). The course of the particular RDFs reveals a varying importance of the particular parts of the CA molecule in binding to CD. More precisely, the furanyl ring plays a dominant role in the case of α- and β-CDs and a more limited one for 2HP-β- and γ-CD. On the other hand, the condensed ring structure of CA (including the lactam moiety) interacts with the inner channel of CDs only in the case of 2HP-β- and γ-CD.

The free energy changes corresponding to the binding of CA by given CD that leads to creating a complex of a 1:1 stoichiometry are equal to: 23.4 ± 1.7 kJ/mol (α-CD-containing complex), 23.1 ± 1.3 kJ/mol (β-CD-containing complex), 38.4 ± 2.7 (γ-CD-containing complex) and 37.9 ± 2.9 (2HP-β-CD-containing complex). Such large values speak for a very high affinity of CA to all types of CDs; the affinity order can be qualitatively expressed as follows: 2HP-β-CD ~ γ-CD > β-CD ~ α-CD. The highest binding affinity is exhibited toward 2HP-β-CD and γ-CDs, which can be ascribed to the largest surface area corresponding to the guest/host contact. As in none of the systems any intensive interactions of the strongly attractive character were observed (e.g., stable and systematic guest–host hydrogen bonding), we concluded that binding is driven primarily by the solvent effect, i.e., the energetically favorable reduction in the area of contact between polar solvent and non-polar parts of the guest and host molecules. Additionally, the contribution of entropic effects is expected to exist, resulting from the alteration of both the conformational properties of guest and host molecules as well as the water mobility.

In general, the binding of CA by various CDs can be considered in a hierarchical manner, such that the number of guest moieties interacting with the cyclodextrin molecule is correlated with its size: the smallest α-CD exhibits direct and intensive contact with the furan group; the methoxy moiety is additionally involved in the case of β-CD, whereas the massive condensed ring structure and its vicinity are exploited in contact with the largest γ-CD as well as with 2HP-β-CD, where additional functional groups are involved. Interestingly, the energetic effect accompanying binding is not straightforwardly correlated with this qualitative observation, probably due to diverse patterns of the guest–host interactions.

## 3. Discussion

Frequently applied spectroscopic techniques can provide essential information on the products obtained with the association of a guest molecule to a CD. This type of mixture leads to a superposition of the two spectra (i.e., originating from guest and CD) without causing any changes in particular spectral contributions. Typically, in a plain inclusion complexation, new bands should not emerge, which may be an indicator of a new chemical bond’s appearance in the resulting product correlating with other interaction types [65].

However, inclusion complexation could lead to remarkable variations in the specific bands of the guest molecule. For instance, the severe decrease or total disappearance of the characteristic bands may indicate the intense guest–host interactions and conceivably inclusion complexation [66], which was observed in this study. For instance, a shift of band attributed to carbonyl (C=O) stretching towards higher frequencies with associated broadening and a decrease in intensity could be connected with the intermolecular hydrogen bond dissociation related to crystalline structures of β-CD [67] in contrast to 2HP-β-CD, which have amorphous structures [68].

In this research, we have confirmed the complexation of CA with all types of studied CDs, which was proven by the disappearance of bands assigned to CH_3_, C–C and C–H groups of CA in the 1600–1500 cm^−1^ range in the CD+CA Raman spectra. Additionally, pronounced changes in the intensity of the ~1346 cm^−1^ band attributed to C–O, C–N, and CH_2_ stretching modes in the furanyl(methoxyimino)acetyl group and ~2944 cm^−1^ band correlated with substituents in the β-lactam ring compared to the spectrum of pure CA were observed.

The bending vibrations of CH_2_OH groups at 1346 cm^−1^ and C–O stretching, and CH in plane bending mode of the aromatic rings and OH in plane bending at 1244 and 1246 cm^−1^ in the complexes of 2HP-β-CD+CA, β-CD+CA and α-CD+CA, respectively, are the evidence of interactions between the hydroxymethyl groups of 2HP-β-CD, hydroxyl groups of α-CD, β-CD and the carbonyl groups of CA [69]. It implies strong restriction of the C=O group inside cyclodextrin’s hollow and is evidence of a basic complexation for the guest molecule incorporated in the torus cavity, as proved in the case of complexes of fentanyl and ibuprofen with β-CD [70]. All CDs+CA samples exhibit a band located at 858–851 cm^−1^ attributed to skeletal and OCH side group deformational vibrations. This band, present at 858 in γ-CD+CA and 854 cm^−1^ in α-CD+CA, shifts to 852 in β-CD+CA and 851 cm^−1^ in 2HP-β-CD+CA, resulting both from the difference in the number of d-glucopyranose units as well as in the character of interactions, and therefore this band has a similar position in the case of β-CD and its derivative. The band at 1157 cm^−1^ is recorded only in the spectrum of γ-CD+CA, which corresponds to the participation of –C=CH functional groups present in the furanyl ring in CA in complexation with the γ-CD cavity [71], but considering all performed analyses, it seems to be a slightly different type of interaction than with α-CD. A much smaller contribution of OH groups in 2HP-β-CD+CA revealed by Raman intensity coefficient calculations results from the presence of fewer OH groups in the inner cavity area than in the case of other CDs and the substitution of the hydroxyl groups by propyl groups [16]. A high intensity band is clearly observed for the C–C–C bending at 496 cm^−1^ for α-CD+CA, and less intensity was recorded for β-CD+CA (497 cm^−1^) and 2HP-β-CD+CA; also, the bands at 1366 (α-CD+CA) and 1370 cm^−1^ (γ-CD+CA) attributed to C-C stretching exhibit a lower intensity as well as increased intensity of C–H stretching (1476 and 1472 cm^−1^ for α-CD+CA and γ-CD+CA, respectively) compared to the other modes, proving a similar type of interactions between CA [45] and α- and γ-CD. Moreover, an increased frequency of stretching vibrational modes of hydroxyl (O–H) groups in α-CD+CA and γ-CD+CA conjugates may imply the hydrogen bonds weakening in the host molecule and hence increasing the amphiphilic properties of CDs, especially in the case of γ-CD possessing the largest surface area corresponding to the guest–host interactions [72]. Molecular dynamics simulation demonstrated that α-CD+CA and γ-CD+CA have the highest intensity of interactions with water molecules, which was also reflected in spectral deconvolution for γ-CD+CA. The 880–820 cm^−1^ band decomposition also revealed small participation of CNC and C–O–C groups in the interactions of β-CD with CA. On the other side, we can state many similarities in molecular structure between α-CD+CA and β-CD+CA, as well as γ-CD+CA and 2HP-β-CD+CA complexes, which was proven by intensity ratio calculations, second derivatives determination and Raman imaging. The resemblance between γ-CD+CA and 2HP-β-CD+CA complexes despite significantly different sizes of cavities was demonstrated.

The direct comparison of the interpreted spectra and the MD simulation results is not straightforward due to a series of factors, including, e.g., the simplified (classical) potential of interactions applied during the MD simulations, the inherently flexible character of the CD+CA complex and non-trivial interpretation of the measured spectra. In spite of these difficulties and uncertainties, there exists a number of findings that are characteristic of both the MD simulations and experimental measurements. More precisely:

1. The most fundamental observation regarding the guest–host type of complexation of CA exploiting the inner cavity of CDs is common for both methods.
2. The contribution of guest–host hydrogen bonding can be identified as originating from the interactions of either hydroxyl (α-CD), both hydroxyl and hydroxymethyl groups (γ-CD) or hydroxypropyl groups (2HP-β-CD) with hydrogen and acceptors located in the CA molecule.
3. The crucial role of the furanyl ring is confirmed in the case of binding by most CDs; additionally, the involvement of the lactam ring is characteristic for γ-CD, which is interpreted in sterical terms.
4. The larger number of CA moieties involved in binding to 2HP-β-CD in comparison to other CDs is confirmed as well.
5. Overall, the MD results explain the differences between binding modes observed for various complexes. Specifically, the similarities of the binding modes exhibited by α-CD and β-CD result from the analogous arrangement of the CA molecule in the binding cavities of CDs, including the interactions with the same furanyl group. The different binding mechanism found for γ-CD originates from the contribution of the condensed ring interacting with the inner cavity of CD. Finally, the spectra for 2HP-β-CD, differing to the largest extent in comparison to those for remaining systems, are the results of reorientation of the CA molecule in the CD binding cavity and the intensive interactions with the hydroxypropyl groups, which are absent in alternative CDs.

Although the correlation of the calculated free energies of binding is rather weakly correlated with the CD-characteristic descriptors given in Table 1, one can note that the corresponding values roughly correspond to the area of the contact between CD and CA molecules. α-CD and β-CD binding is associated with the involvement of a rather small-sized group of CA, namely furanyl and *N*-methoxy groups. The larger cavity of γ-CD allows it to increase the contact by the involvement of a much larger number of CA moieties. Finally, the possibilities of intermolecular guest–host interactions are significantly increased in the case of 2HP-β-CD due to the presence of 2-hydroxypropyl groups, which elongate the apparent dimensions of the CD cavity and facilitate the interactions between non-polar parts of CA and CD.

The most examples of the guest–host type systems with various pharmaceuticals were reported for β-CD, relatively fewer for the smallest α-CD, and even fewer examples were found for the most water-soluble complexes with γ-CD, characterized by the largest hollow size and the highest kinetic lability, which may mean a lower binding strength of the guest molecule [73]. In summary, the study of CA complexation with CDs showed that α-CD with the smallest cavity as well as β-, γ-, and 2HP-β-CDs arrange rotaxane-like structures in which the guest molecule (CA) is threaded through a cyclodextrin host molecule, proving the formation of inclusive type complexes, where both furan and methoxy moieties and interactions with the macrocyclic ring of CDs are involved to varying extents depending on the type of CD. Moreover, some mode of interaction in the case of α- and β-CD inclusion complexes is fairly congenial and probably involves the same chemical groups.

New information obtained from these analyses relates to the elucidation of how individual cyclodextrins form complexes with CA and the identification of particular chemical groups responsible for these interactions. For these purposes, we have used Raman spectroscopy and imaging and molecular dynamics simulations, which complemented each other perfectly. By combining these different approaches, we have obtained a very good agreement between experimental and computational results, acquiring both qualitative and quantitative assessment. Simultaneous use of these two methods was also applied successfully by other researchers in pharmaceutical and biochemical fields. They were utilized inter alia to study the crystal structure of the chiral antiparasitic drug Praziquantel [74], examine the equilibrium geometry, bonding features, and harmonic vibrational frequencies in benzothiazole [75], investigate the polymorphic behavior of crystallized curcumin [76], explore the interaction of hypericin with serum albumins [77], and chromate with 4-(2-mercaptoethyl)pyridinium [78]. In the future, it will be possible to apply these techniques to analyze more types of active compounds in pharmaceutical formulations and guest–host complexes, leading to the design of drugs with appropriate pharmacokinetic properties, thereby increasing their bioavailability.

## 4. Materials and Methods

### 4.1. Preparation of Cefuroxime Axetil Complexes with Cyclodextrins

The uncoated tablets were prepared by mixing the weighed amount of cyclodextrin with the cefuroxime axetil (USP Reference Standard No. 1098220, Merck, Darmstadt, Germany) in the ratio of masses: 1 part of active substance + 2 parts of cyclodextrin (α-, β-, γ, and 2HP-β-CD, respectively). CDs (α-CD, CAS 10016-20-3; β-CD, CAS 7585-39-9; γ-CD CAS 17465-86-0; 2HP-β-CD, CAS 128446-35-5) were obtained from Sigma-Aldrich, Saint Louis, MO, USA. The substitution pattern was deduced from the information obtained from the manufacturer about the molar mass and the extent of labeling. The ingredients were thoroughly mixed in a mortar and then ground for 30 min to obtain a homogeneous mass. The homogeneous mass was compressed and tableted in an EK0 impact tablet press (Korsch-Erweka, Langen, Germany). Flat, triple stamps with a diameter of 3 mm were used, and the punch pressure was set to 30 kN. All procedures were performed at room temperature. The average weight of the tablet was 17 mg and hardness was 0.6 kG. For Raman microspectroscopy, the whole, uncrushed tablets were measured.

### 4.2. Raman Spectroscopy and Imaging

The DXR Raman Microscope (Thermo Scientific, Waltham, MA, USA) was used to record the Raman spectra and chemical maps. The 780 nm excitation laser wavelength and a 12 mW output power were applied. The spectra were registered in the 3100–100 cm^−1^ spectral range, at 4 cm^−1^ of Raman shift resolution. The settings were as follows: an exposure time of 3 s, 10 exposures per point with ×50 objective and 25 μm slit aperture, without photobleaching. Microscope was equipped with CCD Camera (Sentech, Japan) and 0.8 mega-pixel CCD sensor. Mapping consisted of 20 × 20 measure points (400 spectra) with step size of 5 µm. Tight focus on the sample surface (Olympus, Tokyo, Japan) with numerical aperture (NA) was used, leading to the size of laser beam diameter of about 1.6 µm. Samples were investigated at room temperature and the parameters of measurements were optimized in order to avoid sample heating. All data processing and image assembly were performed using OMNIC (ver. 8.2.0.387, Thermo Fisher Scientific, Madison, WI, USA) and CytoSpec (ver 2.00.01, Berlin, Germany) software. The ten spectra from each sample were collected, baseline corrected and then averaged before analysis. In order to evaluate the molecular organization in studied samples, second order derivative spectra were determined by the Savitzky–Golay algorithm with smoothening (seventeen points). To calculate I_OH/CH2_, I_C=O/OH_ and I_C=O/CH2_ intensity ratios, the values were read at the specific Raman shift wavenumbers after normalization to the 1127 cm^−1^ (α-CD+CA and β-CD+CA), 1133 cm^−1^ (γ-CD+CA), and 1126 cm^−1^ bands (2HP-β-CD+CA), assigned to C-O-C stretching mode. The results were obtained from ten spectra, and presented in the form of a table as mean values ± standard deviation (SD). Calculations were carried out using Statistica 13 software (StatSoft Inc., Tulsa, OK, USA). Deconvolution of the overlapping bands in the range of 880–820 cm^−1^ was conducted with the use of mixed Lorentzian/Gaussian peak fitting function in GRAMS/AI software (ThermoGalactic Industries, Waltham, MA, USA), analogously to other described spectral operations.

### 4.3. Molecular Modeling

The compounds under consideration included: CA, α-, β-, γ-CD and 2HP-β-CD. The latter compound was substituted at 6 out of 7 possible 2-OH groups by the hydroxypropyl moieties. Eight independent systems were created, each of them containing both one CD molecule and one CA molecule. The initial arrangements of these two molecules were either random (disconnected molecules of both CA and CD separated by a distance of at least 1.5 nm) or the CA–CD complexes, corresponding to the structures reported in ref. [39]. The molecules were placed in the cubic simulation boxes of dimensions 4.5 × 4.5 × 4.5 nm^3^ and surrounded by ~3000 explicit water molecules (SPC model) [79]. Additionally, for free energy calculations, the systems composed of single CA molecule and water were considered.

All molecular dynamics (MD) simulations were carried out within the GROMACS 2016.4 [80] package. The potential of interactions was taken from the carbohydrate-dedicated force field GROMOS 56A6_CARBO/CARBO_R_ [81,82], validated also in the context of cyclodextrins [83]. The GROMOS parameters for CA were generated by the Automated Topology Builder 3.0 online server [84]. The unbiased simulations were carried out under periodic boundary conditions and in the isothermal-isobaric ensemble. The temperature was maintained close to its reference value (298 K) by applying the V-rescale thermostat [85], whereas for the constant pressure (1 bar, isotropic scaling) the Parrinello–Rahman barostat [86] was used with a relaxation time of 0.4 ps. The equations of motion were integrated with a timestep of 2 fs using the leap-frog scheme [87]. The solute bond lengths were constrained by application of the LINCS procedure [88] with a relative geometric tolerance of 10^−4^. The full rigidity of the water molecules was enforced by application of the SETTLE procedure [89]. The translational center-of-mass motion was removed every timestep separately for the solute and the solvent. The non-bonded interactions were calculated using a single cut-off distance set to 1.4 nm and Verlet list scheme. The reaction-field correction [90] was applied to account for the mean effect of the electrostatic interactions beyond the long-range cut off distance, using a relative dielectric permittivity of 61 as appropriate for the SPC water model [91].

All the systems were pre-optimized by a constant-pressure MD equilibration of duration of 1 ns at 1 bar and 298 K, ensuring an effective solvent density appropriate for these conditions in the subsequent production simulations. After equilibration, all unbiased simulations were carried out for 500 ns and the trajectory was saved every 2 ps.

The free energy differences corresponding to binding of CA by each of the considered CDs were calculated by using the thermodynamic integration (TI) method. The TI perturbations relied on a series of independent MD simulations with gradual modifications of the non-bonded potential corresponding to interactions of CA molecule with its environment. More precisely, the Lennard-Jones and Coulombic interactions were gradually turned off by using the λ parameter, describing the magnitude of introduced alterations (λ = 0: all interactions present; λ = 1: no interactions between CA and the rest of the system). For all perturbations, 21 evenly distributed points (λ = 0, 0.05, 0.1 … 1) were sampled for 100 ns each. Analysis of the 〈∂H/∂λ〉_λ_ curves as well as the error analysis were performed by using the BAR method [92], as implemented in GROMACS. The TI protocol involved both the CD+CA complexes and sole CA molecule in water. In the former case, the TI simulations were initiated from the structure identified as the equilibrium one, on the basis of the previous stage of MD simulations.

In order to confirm the complexation mode identified during MD simulations, we additionally performed a series of guest–host docking simulations. The CA molecules were prepared by using the Avogadro 1.2.0 software [93] and subsequently optimized within the UFF force field (5000 steps, steepest descent algorithm). The flexible, CA molecules were docked into the CD structures found in the PDB database. In the case of 2HP-β-CD, the 2HP moieties were drawn manually by using Avogadro and the complete structure was optimized at the UFF level of theory. The AutoDock Vina software [94] was applied for docking simulations. The procedure of docking was carried out within the cuboid region which covered the whole CD molecule.

## 5. Conclusions

Physicochemical and molecular characteristics obtained by the Raman microspectroscopic method confirmed successful CD+CA complex formation. The complexation of CA with α-, β-, γ-CD, and 2HP-β-CD leads to a remarkable increase in its water solubility and causes a better stabilization and higher bioavailability. The fundamental changes which appeared in the Raman spectra of CD+CA complexes were related mainly to the spectral ranges of C=O and O-H stretching vibrations. These variations indicated an elementary complexation mechanism for the CA molecule included in the CD torus host cavity. The molecular dynamics simulations allowed for a more detailed interpretation of the collected data, including the identification of the particular chemical group involved in the most crucial interactions between CA and CD. Overall, the CD+CA complexes are very flexible and the bound CA molecule can interact with various groups of CD. The most conserved binding mode always involves the inner cavity of CD and either the furanyl (typical for α- and β-CD) or condensed ring of CA (typical for γ-CD and 2HP-β-CD). The binding is driven by solvent-exclusion effects with a small contribution of other types of attractive interactions (mainly hydrogen bonds). The CD+CA affinity order is as follows: 2HP-β-CD ~ γ-CD > β-CD ~ α-CD, and is roughly correlated with the CD+CA contact area.

## Figures and Tables

**Table 1:** Selected physicochemical properties of CDs [15,16,17,18,19].

Cyclodextrin	Number of Glucopyranose Units	Molecular Weight [g/mol]	Cavity Inner Diameter [Å]	Cavity Outer Diameter [Å]	Cavity Height [Å]	Cavity Volume [Å]	Specific Rotation, Optical Activity [α]^D^_20_ [H_2_O, 1%]	Solubility in H_2_O [g/100 mL, 25 °C]
α-CD	6	973	4.7–5.3	14.6	7.9	174	+150.5°	14.5
β-CD	7	1135	6.0–6.5	15.4	7.9	262	+162.5°	1.85
γ-CD	8	1297	7.0–8.3	17.5	7.9	427	+177.4°	23.20
2HP-β-CD	7	1460	6.0	-	-	-	+135.0°	33

**Table 2:** The most significant bands recorded in the Raman spectra of studied CA–CD complexes (assignments were made in accordance with refs. [44,45,46,47]).

Raman Shift/cm^−1^				Band Assignments
Sample				
α-CD+CA	β-CD+CA	γ-CD+CA	2HP-β-CD+CA	
2993	-	-	-	CH_3_ antisymmetric stretching
-	-	2975	2982	CH stretching
2920	-	-	-	
2892	2908	2883	2881	
2956	-	-	-	CH stretching or wagging
-	2939	2932	2931	CH_2_ antisymmetric stretching, CH_3_ symmetric stretching
2918	-	-	-	CH stretching or wagging
-	-	-	2866	CH_2_ symmetric stretching
-	-	2839	2839	CH_3_ stretching
1476	-	1472	-	CH deformational
1456	1449	1453	1456	CH_2_ deformational
-	1414	1400	1403	C–O–C symmetric and antisymmetric stretching
-	1389	-	-	C–H stretching or wagging
1366	-	1370	-	C–C stretching and ring deformation
-	-	-	1346 *	Deformations of the CH_2_OH group, C–O, C–N stretching
1331	1335	1327	1335	CH_2_ deformational
1264	-	1264	1267	C=O stretching
1244	1248	-	-	C=O stretching, CH in plane bending of the aromatic rings, OH in plane bending, CH_2_ stretching
-	-	1157 *	-	–C=C–H antisymmetric angular deformation in plane, furanyl ring in CA
1137	-	1133	1142	C-O-C stretching
1127	1127	-	1126	C–O–C symmetric stretching
-	1111	1109	-	C–O–C symmetric and antisymmetric stretching of glycosidic bonds
1081	1083	-	1082	
1057 1036	1046	1040	1040	C–O stretching
-	1000 *	-	-	“breathing mode” of the aromatic carbon ring, C=C–H stretching
-	-	969 *	-	C–H and C–OH deformational
952	948	948	948	Skeletal mode of α-(1–4) linkage (delocalized mode), C–O stretching
938 864	926 864	917 -	925 -	Glucopyranose (C–O–C) skeletal mode of α-anomers
854	851	858	852	Skeletal vibrations, OCH side group deformational of d-glucopyranose units, CNC symmetric stretching of the imide
843	-	-	-	C–O–C antisymmetric stretching
749	756	757	759	d-glucopyranose ring breathing mode and C–C central stretch
708	710	706	707	C–H out-of-plane bending of CA
609 *	-	-	-	C–C–C ring in-plane bending of CA
-	588	592	590	Symmetric oxygen breathing vibration, C–O bending
570	571	-	-	OH wagging
-	-	513 *	-	In-plane C–C stretching and ring deformation of CA
496	497	-	496	C–C–C bending
479	477	-	478	Skeletal vibrations, amylose
456	439	-	443	CH stretching
416 357	- 363	427 -	- 234	OH stretching
389	-	374	-	C–C stretching
-	321 *		-	External C–OH out of plane bending of glucopyranose units
310	-	302	-	C–C antisymmetric stretching
286	-	272	-	C–O stretching
153 *	-	-	-	Breathing motions of oxygen atoms in the macrocyclic ring
138 *	-	-	-	Stretching or bending vibrations of hydrogen bonds

Asterisks (*) indicate the most significant differences between the samples.

**Table 3:** The comparison of Raman intensity ratios for specific chemical groups. The calculated results are demonstrated as mean ± standard deviation (SD).

The Raman Intensity Ratio	Sample			
	α-CD+CA *	β-CD+CA **	γ-CD+CA ***	2HP-β-CD+CA ****
I_C=O/CH2_	0.249 ± 0.055	0.330 ± 0.056	0.394 ± 0.100	0.238 ± 0.081
I_OH/CH2_	0.492 ± 0.081	0.574 ± 0.084	0.495 ± 0.133	0.132 ± 0.086
I_C=O/OH_	0.505 ± 0.094	0.588 ± 0.138	0.837 ± 0.250	1.770 ± 0.990

* C=O stretching 1264 cm^−1^, CH_2_ deformational 1331 cm^−1^, OH stretching 357 cm^−1^; ** C=O stretching 1248 cm^−1^, CH_2_ deformational 1335 cm^−1^, OH stretching 363 cm^−1^; *** C=O stretching 1264 cm^−1^, CH_2_ deformational 1327 cm^−1^, OH stretching 427 cm^−1^; **** C=O stretching 1267 cm^−1^, CH_2_ deformational 1335 cm^−1^, OH stretching 234 cm^−1^.

## Data Availability

The data presented in this study are available on request from the corresponding author.

## Abbreviations

CD	cyclodextrin
α-CD	α-cyclodextrin
β-CD	β-cyclodextrin
γ-CD	γ-cyclodextrin
CA	cefuroxime axetil
2HP-β-CD	2-hydroxypropyl-β cyclodextrin

## References

[1] Crini G. (2014). Review: A History of Cyclodextrins. Chem. Rev..

[2] Khalid Q., Ahmad M., Minhas M.U. (2017). Synthesis of β-cyclodextrin hydrogel nanoparticles for improving the solubility of dexibuprofen: Characterization and toxicity evaluation. Drug Dev. Ind. Pharm..

[3] Wimmer T. (2012). Cyclodextrins. ULLMANN’S Encyclopedia of Industrial Chemistry.

[4] Wimmer R., Aachmann F.L., Larsen K.L., Petersen S.B. (2002). NMR diffusion as a novel tool for measuring the association constant between cyclodextrin and guest molecules. Carbohydr. Res..

[5] Kurkov S.V., Ukhatskaya E.V., Loftsson T. (2011). Drug/cyclodextrin: Beyond inclusion complexation. J. Incl. Phenom. Macrocycl. Chem..

[6] Brewster M.E., Loftsson T. (2007). Cyclodextrins as pharmaceutical solubilizers. Adv. Drug Deliv. Rev..

[7] Jin L., Liu Q., Sun Z., Ni X., Wei M. (2010). Preparation of 5-fluorouracil/β-cyclodextrin complex intercalated in layered double hydroxide and the controlled drug release properties. Ind. Eng. Chem. Res..

[8] Duchene D., Wouessidjewe D., Denis W. (1992). Industrial Uses of Cyclodextrins and Their Derivatives. J. Coord. Chem..

[9] Braga S.S. (2019). Cyclodextrins: Emerging Medicines of the New Millennium. Biomolecules.

[10] Loftsson T., Jarho P., Másson M., Järvinen T. (2005). Cyclodextrins in drug delivery. Expert Opin. Drug Deliv..

[11] Tiwari G., Tiwari R., Rai A. (2010). Cyclodextrins in delivery systems: Applications. J. Pharm. Bioallied Sci..

[12] Stella V.J., He Q. (2008). Cyclodextrins. Toxicol. Pathol..

[13] Irie T., Uekama K. (1997). Pharmaceutical Applications of Cyclodextrins. III. Toxicological Issues and Safety Evaluation. J. Pharm. Sci..

[14] Vyas A., Saraf S.S., Saraf S.S. (2008). Cyclodextrin based novel drug delivery systems. J. Incl. Phenom. Macrocycl. Chem..

[15] Saokham P., Muankaew C., Jansook P., Loftsson T. (2018). Solubility of Cyclodextrins and Drug/Cyclodextrin Complexes. Molecules.

[16] Yong C.W., Washington C., Smith W. (2008). Structural Behaviour of 2-Hydroxypropyl-β-Cyclodextrin in Water: Molecular Dynamics Simulation Studies. Pharm. Res..

[17] Miranda J.C., Martins T.E.A., Veiga F., Ferraz H.G. (2011). Cyclodextrins and ternary complexes: Technology to improve solubility of poorly soluble drugs. Braz. J. Pharm. Sci..

[18] Jansook P., Ogawa N., Loftsson T. (2018). Cyclodextrins: Structure, physicochemical properties and pharmaceutical applications. Int. J. Pharm..

[19] Jambhekar S.S., Breen P. (2016). Cyclodextrins in pharmaceutical formulations I: Structure and physicochemical properties, formation of complexes, and types of complex. Drug Discov. Today.

[20] Uekama K., Hirayama F., Irie T. (1998). Cyclodextrin Drug Carrier Systems. Chem. Rev..

[21] Malanga M., Szemán J., Fenyvesi É., Puskás I., Csabai K., Gyémánt G., Fenyvesi F., Szente L. (2016). “Back to the Future”: A New Look at Hydroxypropyl Beta-Cyclodextrins. J. Pharm. Sci..

[22] Miyake K., Arima H., Hirayama F., Yamamoto M., Horikawa T., Sumiyoshi H., Noda S., Uekama K. (2000). Improvement of Solubility and Oral Bioavailability of Rutin by Complexation with 2-Hydroxypropyl-β-cyclodextrin. Pharm. Dev. Technol..

[23] Gould S., Scott R.C. (2005). 2-Hydroxypropyl-β-cyclodextrin (HP-β-CD): A toxicology review. Food Chem. Toxicol..

[24] Mohit V., Harshal G., Neha D., Vilasrao K., Rajashree H. (2010). Effect of preparation method on complexation of Cefdinir with β-cyclodextrin. J. Incl. Phenom. Macrocycl. Chem..

[25] Leder R.D., Carson D.S. (1997). Cefuroxime Axetil (Ceftin®): A Brief Review. Infect. Dis. Obstet. Gynecol..

[26] Harding S.M., Williams P.E., Ayrton J. (1984). Pharmacology of Cefuroxime as the 1-acetoxyethyl ester in volunteers. Antimicrob. Agents Chemother..

[27] Chaudhry S.B., Veve M.P., Wagner J.L. (2019). Cephalosporins: A Focus on Side Chains and β-Lactam Cross-Reactivity. Pharmacy.

[28] Ginsburg C.M., McCracken G.H., Petruska M., Olson K. (1985). Pharmacokinetics and bactericidal activity of cefuroxime axetil. Antimicrob. Agents Chemother..

[29] Pichichero M.E. (2007). Use of selected cephalosporins in penicillin-allergic patients: A paradigm shift. Diagn. Microbiol. Infect. Dis..

[30] Sader H.S., Jacobs M.R., Fritsche T.R. (2007). Review of the spectrum and potency of orally administered cephalosporins and amoxicillin/clavulanate. Diagn. Microbiol. Infect. Dis..

[31] Jun S.W., Kim M.-S., Jo G.H., Lee S., Woo J.S., Park J.-S., Hwang S.-J. (2010). Cefuroxime axetil solid dispersions prepared using solution enhanced dispersion by supercritical fluids. J. Pharm. Pharmacol..

[32] Shah M., Shah V., Ghosh A., Zhang Z., Minko T. (2015). Molecular Inclusion Complexes of β-Cyclodextrin Derivatives Enhance Aqueous Solubility and Cellular Internalization of Paclitaxel: Preformulation and In vitro Assessments. J. Pharm. Pharmacol..

[33] Loftsson T. (2002). Cyclodextrins and the Biopharmaceutics classification system of drugs. J. Incl. Phenom. Macrocycl. Chem..

[34] Skoog D.A., Holler F.J., Croush S.R. (2007). Principles of Instrumental Analysis.

[35] Rohman A., Windarsih A., Lukitaningsih E., Rafi M., Betania K., Fadzillah N.A. (2020). The use of FTIR and Raman spectroscopy in combination with chemometrics for analysis of biomolecules in biomedical fluids: A review. Biomed. Spectrosc. Imaging.

[36] Gordon K.C., McGoverin C.M. (2011). Raman mapping of pharmaceuticals. Int. J. Pharm..

[37] Khan G.M., Wazir F., Zhu J.B. (2001). Ibuprofen-Cyclodextrin Inclusion Complexes: Evaluation of Different Complexation Methods. J. Med. Sci..

[38] Mizera M., Szymanowska D., Stasiłowicz A., Siąkowska D., Lewandowska K., Miklaszewski A., Plech T., Tykarska E., Cielecka-Piontek J. (2019). Computer-Aided Design of Cefuroxime Axetil/Cyclodextrin System with Enhanced Solubility and Antimicrobial Activity. Biomolecules.

[39] Sapte S., Pore Y. (2016). Inclusion complexes of cefuroxime axetil with β-cyclodextrin: Physicochemical characterization, molecular modeling and effect of l-arginine on complexation. J. Pharm. Anal..

[40] Shah M., Pore Y., Dhawale S., Burade K., Kuchekar B. (2013). Physicochemical characterization of spray dried ternary micro-complexes of cefuroxime axetil with hydroxypropyl-β-cyclodextrin. J. Incl. Phenom. Macrocycl. Chem..

[41] Talaczynska A., Mizera M., Szybowicz M., Nowicka A.B., Garbacki P., Paczkowska M., Zalewski P., Kozak M., Oszczapowicz I., Jelinska A. (2016). Studies of the crystalline form of cefuroxime axetil: Implications for its compatibility with excipients. Acta Pol. Pharm..

[42] Talaczyńska A., Lewandowska K., Jelińska A., Garbacki P., Podborska A., Zalewski P., Oszczapowicz I., Sikora A., Kozak M., Cielecka-Piontek J. (2015). Application of Vibrational Spectroscopy Supported by Theoretical Calculations in Identification of Amorphous and Crystalline Forms of Cefuroxime Axetil. Sci. World J..

[43] Venuti V., Crupi V., Fazio B., Majolino D., Acri G., Testagrossa B., Stancanelli R., De Gaetano F., Gagliardi A., Paolino D. (2019). Physicochemical Characterization and Antioxidant Activity Evaluation of Idebenone/Hydroxypropyl-β-Cyclodextrin Inclusion Complex. Biomolecules.

[44] de Oliveira V.E., Almeida E.W.C., Castro H.V., Edwards H.G.M., Dos Santos H.F., de Oliveira L.F.C. (2011). Carotenoids and β-Cyclodextrin Inclusion Complexes: Raman Spectroscopy and Theoretical Investigation. J. Phys. Chem. A.

[45] Tijunelyte I., Dupont N., Milosevic I., Barbey C., Rinnert E., Lidgi-Guigui N., Guenin E., de la Chapelle M.L. (2017). Investigation of aromatic hydrocarbon inclusion into cyclodextrins by Raman spectroscopy and thermal analysis. Environ. Sci. Pollut. Res..

[46] Barron L.D., Gargaro A.R., Wen Z.Q., MacNicol D.D., Butters C. (1990). Vibrational Raman optical activity of cyclodextrins. Tetrahedron Asymmetry.

[47] De Gelder J., De Gussem K., Vandenabeele P., Moens L. (2007). Reference database of Raman spectra of biological molecules. J. Raman Spectrosc..

[48] Paczkowska M., Szymanowska-Powałowska D., Mizera M., Siąkowska D., Błaszczak W., Piotrowska-Kempisty H., Cielecka-Piontek J. (2019). Cyclodextrins as multifunctional excipients: Influence of inclusion into β-cyclodextrin on physicochemical and biological properties of tebipenem pivoxil. PLoS ONE.

[49] Fan D., Ma W., Wang L., Huang J., Zhao J., Zhang H., Chen W. (2012). Determination of structural changes in microwaved rice starch using Fourier transform infrared and Raman spectroscopy. Starch Stärke.

[50] Fechner P.M., Wartewig S., Kleinebudde P., Neubert R.H.H. (2005). Studies of the retrogradation process for various starch gels using Raman spectroscopy. Carbohydr. Res..

[51] Egyed O. (1990). Spectroscopic studies on β-cyclodextrin. Vib. Spectrosc..

[52] Wiercigroch E., Szafraniec E., Czamara K., Pacia M.Z., Majzner K., Kochan K., Kaczor A., Baranska M., Malek K. (2017). Raman and infrared spectroscopy of carbohydrates: A review. Spectrochim. Acta A. Mol. Biomol. Spectrosc..

[53] França de Sá S., Ferreira J.L., Matos A.S., Macedo R., Ramos A.M. (2016). A new insight into polyurethane foam deterioration-The use of Raman microscopy for the evaluation of long-term storage conditions. J. Raman Spectrosc..

[54] Morzyk-Ociepa B., Nowak M.J., Michalska D. (2004). Vibrational spectra of 1-methylthymine: Matrix isolation, solid state and theoretical studies. Spectrochim. Acta Part A Mol. Biomol. Spectrosc..

[55] Podstawka E., Światłowska M., Borowiec E., Proniewicz L.M. (2007). Food additives characterization by infrared, Raman, and surface-enhanced Raman spectroscopies. J. Raman Spectrosc..

[56] Bauer A.J.R. (2014). Raman Spectroscopic Study of Sugars in Common Liquid Sweeteners. Spectroscopy.

[57] Cozar O., Cioica N., Coţa C., Nagy E.M., Fechete R. Plasticizers effect on native biodegradable package materials. Proceedings of the Tim15-16 Physics Conference.

[58] Vázquez M., Oliva M., Téllez-Luis S.J., Ramírez J.A. (2007). Hydrolysis of sorghum straw using phosphoric acid: Evaluation of furfural production. Bioresour. Technol..

[59] Madan J., Dhiman N., Parmar V.K., Sardana S., Bharatam P.V., Aneja R., Chandra R., Katyal A. (2010). Inclusion complexes of noscapine in β-cyclodextrin offer better solubility and improved pharmacokinetics. Cancer Chemother. Pharmacol..

[60] Saha S., Roy A., Roy K., Roy M.N. (2016). Study to explore the mechanism to form inclusion complexes of β-cyclodextrin with vitamin molecules. Sci. Rep..

[61] Esmonde-White K. (2014). Raman Spectroscopy of Soft Musculoskeletal Tissues. Appl. Spectrosc..

[62] Sergeeva A.V., Zhitova E.S., Nuzhdaev A.A., Zolotarev A.A., Bocharov V.N., Ismagilova R.M. (2020). Infrared and Raman Spectroscopy of Ammoniovoltaite, (NH4)2Fe2+5Fe3+3Al(SO4)12(H2O)18. Minerals.

[63] Chan J.W., Taylor D.S., Zwerdling T., Lane S.M., Ihara K., Huser T. (2006). Micro-Raman Spectroscopy Detects Individual Neoplastic and Normal Hematopoietic Cells. Biophys. J..

[64] Chylińska M., Szymańska-Chargot M., Zdunek A. (2014). Imaging of polysaccharides in the tomato cell wall with Raman microspectroscopy. Plant Methods.

[65] Figueiras A., Ribeiro L., Vieira M.T., Veiga F. (2007). Preparation and physicochemical characterization of omeprazole:methyl-beta-cyclodextrin inclusion complex in solid state. J. Incl. Phenom. Macrocycl. Chem..

[66] Sinha V.R., Anitha R., Ghosh S., Nanda A., Kumria R. (2005). Complexation of celecoxib with β-cyclodextrin: Characterization of the interaction in solution and in solid state. J. Pharm. Sci..

[67] Badr-Eldin S.M., Elkheshen S.A., Ghorab M.M. (2008). Inclusion complexes of tadalafil with natural and chemically modified β-cyclodextrins. I: Preparation and In-Vitro evaluation. Eur. J. Pharm. Biopharm..

[68] Kreaz R.M., Dombi G.Y., Kata M. (1996). Increasing The Solubility of Furosemide with Cyclodextrins. Proceedings of the Eighth International Symposium on Cyclodextrins.

[69] Yang Z., Chai K., Ji H. (2011). Selective inclusion and separation of cinnamaldehyde and benzaldehyde by insoluble β-cyclodextrin polymer. Sep. Purif. Technol..

[70] Bratu I., Hernanz A., Gavira J.M., Bora G.H. (2005). FT-IR spectroscopy of inclusion complexes of β-cyclodextrin with fenbuten and ibuprofen. Rom. J. Phys..

[71] Mangolim C.S., Moriwaki C., Nogueira A.C., Sato F., Baesso M.L., Neto A.M., Matioli G. (2014). Curcumin–β-cyclodextrin inclusion complex: Stability, solubility, characterisation by FT-IR, FT-Raman, X-ray diffraction and photoacoustic spectroscopy, and food application. Food Chem..

[72] Witte F., Hoffmann H. (1996). Aggregation Behavior of Hydrophobically Modified β-Cyclodextrins In Aqueous Solution. Proceedings of the Eighth International Symposium on Cyclodextrins.

[73] Challa R., Ahuja A., Ali J., Khar R.K. (2005). Cyclodextrins in drug delivery: An updated review. AAPS PharmSciTech.

[74] Borrego-Sánchez A., Hernández-Laguna A., Sainz-Díaz C.I. (2017). Molecular modeling and infrared and Raman spectroscopy of the crystal structure of the chiral antiparasitic drug Praziquantel. J. Mol. Model..

[75] Sathyanarayanmoorthi V., Karunathan R., Kannappan V. (2013). Molecular Modeling and Spectroscopic Studies of Benzothiazole. J. Chem..

[76] Prasad R., Gupta K.M., Poornachary S.K., Dalvi S.V. (2020). Elucidating the polymorphic behavior of curcumin during antisolvent crystallization: Insights from Raman spectroscopy and molecular modeling. Cryst. Growth Des..

[77] Miskovsky P., Hritz J., Sanchez-Cortes S., Fabriciova G., Ulicny J., Chinsky L. (2007). Interaction of Hypericin with Serum Albumins: Surface-enhanced Raman Spectroscopy, Resonance Raman Spectroscopy and Molecular Modeling Study. Photochem. Photobiol..

[78] Mosier-Boss P.A., Lieberman S.H. (2003). Surface-Enhanced Raman Spectroscopy (SERS) and Molecular Modeling of the Chromate Interaction with 4-(2-Mercaptoethyl)Pyridinium. Langmuir.

[79] Berendsen H.J.C., Postma J.P.M., van Gunsteren W.F., Hermans J. (1981). Interaction Models for Water in Relation to Protein Hydration. Intermolecular Forces.

[80] Abraham M.J., Murtola T., Schulz R., Páll S., Smith J.C., Hess B., Lindah E. (2015). Gromacs: High performance molecular simulations through multi-level parallelism from laptops to supercomputers. SoftwareX.

[81] Hansen H.S., Hünenberger P.H. (2011). A reoptimized GROMOS force field for hexopyranose-based carbohydrates accounting for the relative free energies of ring conformers, anomers, epimers, hydroxymethyl rotamers, and glycosidic linkage conformers. J. Comput. Chem..

[82] Plazinski W., Lonardi A., Hünenberger P.H. (2016). Revision of the GROMOS 56A6 CARBO force field: Improving the description of ring-conformational equilibria in hexopyranose-based carbohydrates chains. J. Comput. Chem..

[83] Gebhardt J., Kleist C., Jakobtorweihen S., Hansen N. (2018). Validation and Comparison of Force Fields for Native Cyclodextrins in Aqueous Solution. J. Phys. Chem. B.

[84] Stroet M., Caron B., Visscher K.M., Geerke D.P., Malde A.K., Mark A.E. (2018). Automated Topology Builder Version 3.0: Prediction of Solvation Free Enthalpies in Water and Hexane. J. Chem. Theory Comput..

[85] Bussi G., Donadio D., Parrinello M. (2007). Canonical sampling through velocity rescaling. J. Chem. Phys..

[86] Parrinello M., Rahman A. (1981). Polymorphic transitions in single crystals: A new molecular dynamics method. J. Appl. Phys..

[87] Hockney R.W. (1970). The potential calculation and some applications. Methods Comput. Phys..

[88] Hess B. (2008). P-LINCS: A Parallel Linear Constraint Solver for Molecular Simulation. J. Chem. Theory Comput..

[89] Miyamoto S., Kollman P.A. (1992). Settle: An analytical version of the SHAKE and RATTLE algorithm for rigid water models. J. Comput. Chem..

[90] Barker J.A., Watts R.O. (1973). Monte Carlo studies of the dielectric properties of water-like models. Mol. Phys..

[91] Heinz T.N., van Gunsteren W.F., Hünenberger P.H. (2001). Comparison of four methods to compute the dielectric permittivity of liquids from molecular dynamics simulations. J. Chem. Phys..

[92] Bennett C.H. (1976). Efficient estimation of free energy differences from Monte Carlo data. J. Comput. Phys..

[93] Hanwell M.D., Curtis D.E., Lonie D.C., Vandermeersch T., Zurek E., Hutchison G.R.J. (2012). Avogadro: An advanced semantic chemical editor, visualization and analysis platform. Cheminformatics.

[94] Trott O., Olson A.J. (2010). AutoDock Vina: Improving the speed and accuracy of docking with a new scoring function, efficient optimization and multithreading. J. Comput. Chem..

